# An Account of a Remarkable Cure of a Dropsy of the Belly, after the Patient Had Been Tapped Sixteen Times

**Published:** 1786

**Authors:** R. Cook

**Affiliations:** Surgeon at Barking, in Essex


XI. *An Account of a remarkable Cure of a Dropfy of the Belly, after the patient had been tapped fixteen times. By Mr.* R. Cook, *Surgeon at Barking, in Effex. Communicated in a Letter to* William Ofborn, *M. D. Phyfician in London, and by him to Dr.* Simmons.

A Widow Lady of Croxley Green, near Rickmanfworth, in Hertfordfhire, about forty years of age, in the beginning of the year 1778, had fymptoms of a dropfy of the belly, which induced her to feek for medical advice; but without effect, as fhe was obliged to undergo the operation of the paracentefis in April 1779, when eleven quarts of water were difcharged.

After

After this, ſhe applied at different times to different practitioners, and among others to the late Dr. Hunter, but her complaint reſiſted every attempt to relieve it; and between April 1779, and March 1785, ſhe ſubmitted to the operation ſixteen times, and loſt above eighty-two gallons of water. Soon after the laſt time of her being tapped, the abdomen was perceived to fill again as uſual; and at this period of the diſeaſe ſhe firſt applied to me. Her general health was now greatly impaired, and her ſpirits much depreſſed; but as the belly was much diſtended, I adviſed her to ſubmit again to be tapped as uſual, and afterwards to adopt the plan I ventured to recommend as the moſt likely, in my opinion, to prevent a return of the complaint. To this ſhe conſented, and ſoon after finding the abdomen uncommonly diſtended, was again about to have recourſe to the operation; but happening one day to be making her bed for the ſake of a little exerciſe, which I had adviſed her to take, ſhe had a ſudden inclination to make water, of which ſhe voided a conſiderable quantity at that time, and in the courſe of about four days upwards of ſix gallons of urine were diſcharged.

She now, by my advice, took calomel in ſmall doſes at bed time, for ſeveral nights, with a ſolution of Glauber's ſalt, in the morning, occaſionally, to prevent the calomel from affecting the mouth. After this I recommended a ſtrong decoction of the Peruvian bark, as a tonic, and twice a day three of the pills mentioned in the note *. Under this courſe, with a proper attention to diet, ſhe ſpeedily recovered her health and ſpirits, and has remained perfectly well ever ſince.

